# Drug Prescriptions in the Outpatient Management of COVID-19: Evidence-Based Recommendations Versus Real Practice

**DOI:** 10.3389/fphar.2022.825479

**Published:** 2022-03-24

**Authors:** Valeria Belleudi, Marco Finocchietti, Filomena Fortinguerra, Aurora Di Filippo, Francesco Trotta, Marina Davoli, Antonio Addis

**Affiliations:** ^1^ Department of Epidemiology, Lazio Regional Health Service, Rome, Italy; ^2^ Italian Medicines Agency (AIFA), Rome, Italy

**Keywords:** COVID-19, outpatient care, evidence-based medicine, drug prescription, drug monitoring

## Abstract

**Background:** Evidence-based recommendations for outpatient management of COVID-19 were published by the Italian Medicines Agency (AIFA) to limit the use of off-label treatments. The aim of this study is to measure the use of outpatient drug treatments in a COVID-19-positive population, taking into account the Italian regulatory agency’s advices.

**Methods:** A descriptive observational study was conducted. All patients testing positive for COVID-19 residing in Lazio region, Italy, with diagnosis date between March 2020 and May 2021 were selected, and outpatient medicine prescription patterns were identified.

**Results:** Independent of AIFA recommendations, the use of drug therapy in the management of outpatient COVID-19 cases was frequent (about one-third of the cases). The most used drug therapy was antibiotics, specifically azithromycin, despite the negative recommendation of AIFA, while the use of corticosteroids increased after the positive recommendation of regulatory agency for the use in subjects with severe COVID-19 disease. The use of hydroxychloroquine was limited to the early pandemic period where evidence on its potential benefit was controversial. Antithrombotics were widely used in outpatient settings, even if their use was recommended for hospitalized patients.

**Conclusion:** In this study, we show a frequent use of drug therapy in the management of outpatient cases of COVID-19, mainly attributable to antibiotics use. Our research highlights the discrepancy between recommendations for care and clinical practice and the need for strategies to bridge gaps in evidence-informed decision-making.

## Introduction

Italy was the first European country able to detect coronavirus disease-19 (COVID-19) in individuals and rapidly turned into one of the most affected areas in the world (Ammassari et al., 2021). As of 15 March 2020, there was more than 22.500 reported cases and 1.625 deaths in the country, and this was the beginning of one of the largest and most serious clusters of COVID-19 in the world (Livingston and Bucher, 2020). At the outbreak of such an epidemic, rumors raised by media, non-peer-reviewed articles, and small clinical trials led physicians to prescribe a vast number of off-label therapies. In other words, clinicians looked for medicines ready to be used, independently of the level of evidence available, supported by high expectations on their potential benefit. Since the beginning of the outbreak, the treatment of COVID-19 including antivirals, chloroquine/hydroxychloroquine, antibiotics, non-steroidal anti-inflammatory drugs (NSAIDs), antithrombotics, corticosteroids, and many unjustified other medications such as biologics, high-dose vitamin C, and vitamin D were recommended and even prescribed in patients with COVID-19 (Barlo et al., 2020; Shojaei and Salari, 2020). However, none of these indications were approved, and they are administered as off-label medications. Most of the recommendations published during the first pandemic stage lack randomized clinical trials evidence for drug treatments with either suspected or confirmed COVID-19 patients in the outpatient setting (Sanders et al., 2020).

Most of the COVID-19-positive patients were asymptomatic or with mild or moderate symptoms and did not necessitate hospitalization. However, clinical evidence on outpatient management in the early stage of the infection is still scarce, and several factors can influence the clinician choice such as sociodemographic and professional factors and perceived disease severity (Martínez-Sanz et al., 2020). Moreover, media coverage and governmental messages about particular pharmacological therapies during the several phases of the pandemic influenced public opinion to consider these drugs resolutive or dangerous to treat COVID-19 (Tuccori et al., 2020).

**FIGURE 1:**
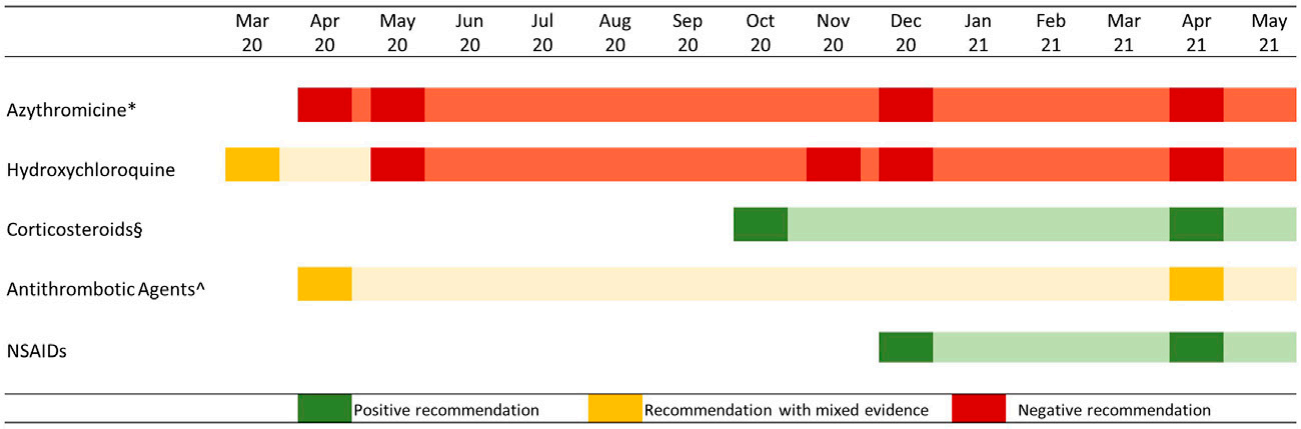
AIFA recommendations on the use of specific drug categories in the outpatient treatment of COVID 19 cases. Note: Attenuated colors show information on card validity, in case of update the diagram reported a new rectangle. * From December 2020 onward, the negative recommendation regarded all antibiotic agents; § Corticosteroid use was recommended in subjects with severe COVID-19 disease mostly combined with oxygen therapy; ^ Antithrombotic use was recommended in hospitalized patients, while outpatient use was controversial.

To overcome this gap, the Italian Medicines Agency (AIFA), in collaboration with scientific committee, published a two-page card on specific outpatient drug treatments that included the rationale, what data supporting its use, with a short methodological analysis, and the use restrictions, or safety concerns (Addis et al., 2020). The agency also issued a warning against the routine use of some of these drugs and their combinations, except in clinical trials, thus bridging clinical studies and clinical practice (Figure 1). The information cards were publicly available on the AIFA’s website and periodically updated with any emerging evidence (Italian Medicines Agency (AIFA), 2021).

Moreover, vitamin D use in COVID-19 patients was suggested by the Italian Society of General Medicine and Primary Care (SIMG), although AIFA did not consider it for outpatient treatment because of limited evidence (The Lancet Diabetes Endocrinology, 2021).

The aim of this study was to measure the use of outpatient drug treatments in COVID-19-positive patients in a real practice setting, taking into account the Evidence-Based Medicine (EBM) recommendations issued by a central competent authority.

## Methods

Lazio region, with a land area of 17,242 km^2^, is one of the 20 administrative regions of Italy and is in the central peninsular area of the country. It has 5,864,321 inhabitants, most of them residents in the metropolitan city of Rome.

This is an observational study based on regional COVID-19 surveillance and drug dispensing registries. The COVID-19 surveillance system was established at the end of February 2020 by the Italian Ministry of Health for reporting cases of SARS-CoV-2 infection and monitoring the evolution of the epidemiological situation. Data on laboratory-confirmed SARS-CoV-2 infections, by a molecular testing method known as reverse real-time PCR, are provided on a daily basis to the national level by all Italian regions. The information used in the surveillance system comes from a complex data flow starting at the local level, aiming to track all COVID-19 cases, both symptomatic and asymptomatic.

The regional drug dispensing registry is limited to drugs reimbursed by the healthcare system dispensed from public and private pharmacies and by hospital at discharge, so we cannot track the in-hospital drug use (e.g., tocilizumab and antiviral drugs). Drugs are identified by the national drug register code, which refers to the International Anatomical Therapeutic Chemical Classification System (ATC).

All COVID-19-positive patients residing in Lazio region with diagnosis dates between 1 March 2020 and 24 May 2021 were selected, and an outpatient medicine prescription pattern was identified. In particular, for each patient, all medicines prescribed in the period from 3 days before to 7 days post-diagnosis were extracted, and the following drug classes were considered: antibiotics (including azithromycin), corticosteroids (including dexamethasone), antithrombotic agents (including heparins), NSAIDs, oxygen, vitamin D, and hydroxychloroquine.

For each study drug, the monthly prevalence of use was calculated considering the percentage of individuals with at least one prescription for investigated drugs on the total of monthly COVID-19 cases, and its 95% confidence intervals (CI) were estimated. Moreover, the monthly ranking of the top 10 COVID-19 outpatient therapies was analyzed and a bump chart was created.

## Results

**FIGURE 2:**
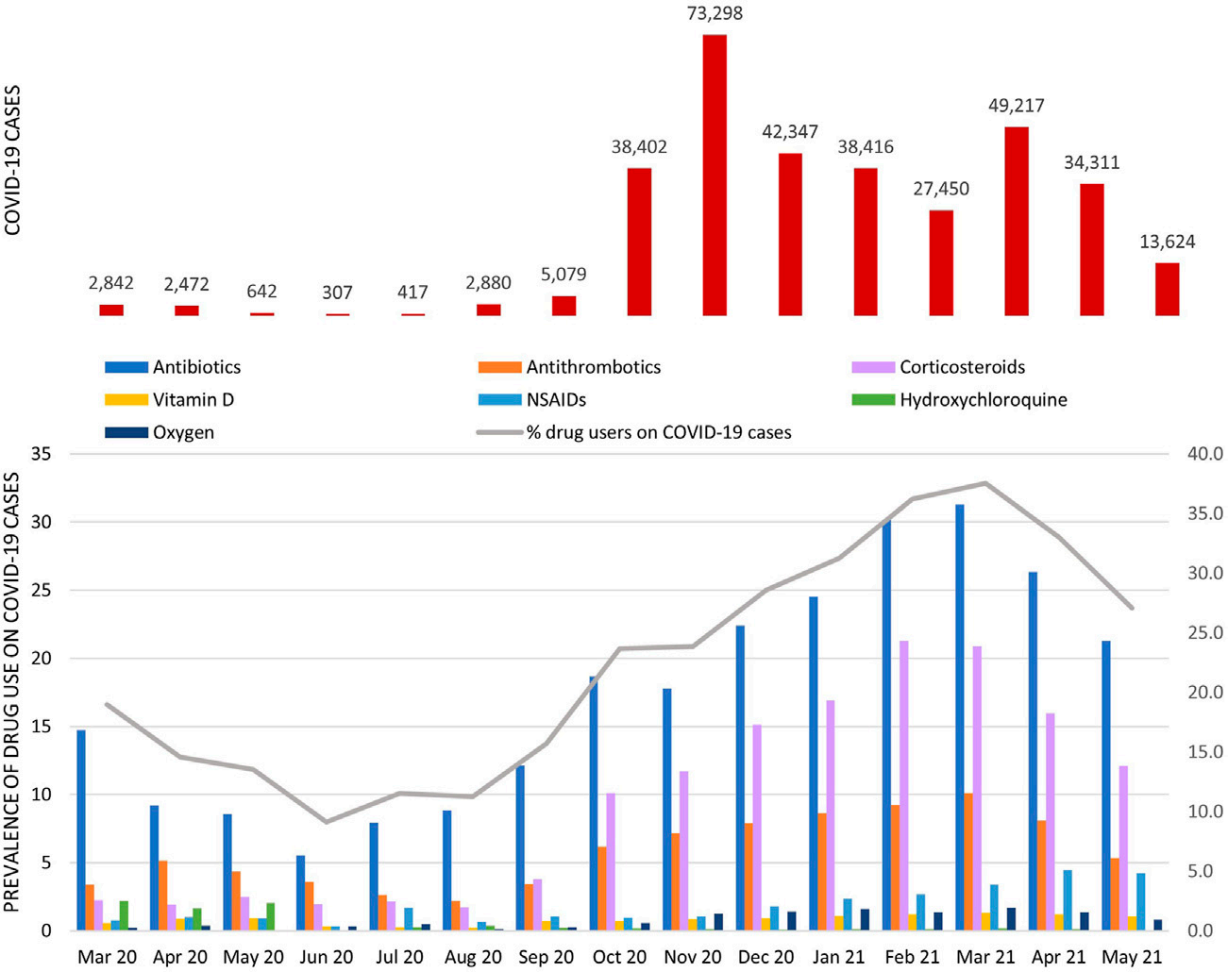
COVID-19 cases and prevalence of study drug use among COVID-19 cases stratified by calendar month. The gray line represents the monthly percentage of COVID-19 cases with at least one study drug in the exposure window (from 3 days before to 7 days post-COVID-19); bars show the monthly prevalence of use of specific drug classes.

In Lazio region (about six million inhabitants), in the study period, 331.704 COVID-19 cases were selected, of which 29% had at least one prescription of study drug. The monthly percentage of drug users ranged from 11.3% (95% CI: 9.7–12.8%) in August 2020 to 37.6% (95% CI: 36.9–38.2%) in March 2021 (Figure 2; for details, see Supplementary Table S1).

Among patients with SARS-CoV-2 infection, the prevalence of antibiotic use was the highest in all months considered, particularly from October 2020 onward, the percentage exceeded 17.5% [minimum 17.8% (95% CI: 17.5–18.1%) in November 2020; maximum 31.3% (95% CI: 30.9–31.7%) in March 2021], of which about 70% was attributable to azithromycin use (Supplementary Table S2).

**FIGURE 3:**
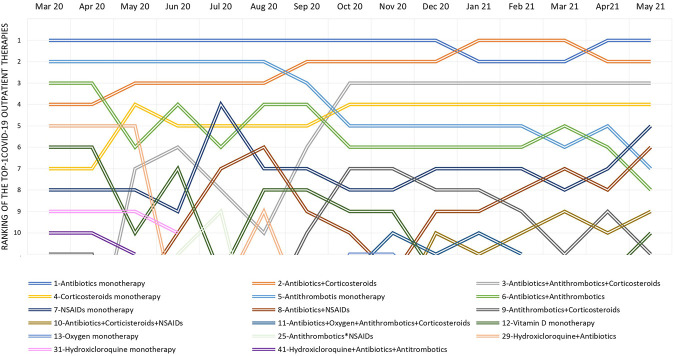
Ranking of the top 10 COVID-19 outpatient therapies during the period from March 2020 to February 2021 and variation by calendar month. Note: In the legend, COVID-19 outpatient therapy and the rank observed considering the entire study period are reported.

The monthly prevalence of antithrombotic agent use had a fluctuating trend, with a clear increase in the period from December 2020 to April 2021 (mean monthly prevalence in this period equal to 8.8%). In this drug class, the overall percentage attributable to heparin use was equal to 79% (Supplementary Table S2). From October 2020 onward, there was an increase in the monthly prevalence of corticosteroid use with a pick of 21.3% (95% CI: 20.8–21.8%) observed in February 2021. Over the study period, vitamin D use remained almost stable with an average monthly prevalence of use equal to 1%, while NSAID use increased from December 2020 onward. Hydroxychloroquine use was very low overall (prevalence of use <0.5%), with a pick in the period from March to May 2020 (mean monthly prevalence of 2%). The monthly prevalence of oxygen use increased from November 2020 onward and reached a mean monthly prevalence of 1.3% compared to 0.3% observed in the previous period. The ranking of the top ten COVID-19 outpatient therapies varied over time (Figure 3).

Considering the entire study period, the top three therapies were based on the antibiotic agent used as monotherapy (first), combined with corticosteroid (second), and combined with both corticosteroids and antithrombotics (third). Specifically, the second rank of the combination antibiotic + corticosteroid was reached as of September 2020, while the third rank of the triple therapy, antibiotic + corticosteroid + antithrombotic, was reached as of October 2020 (in line with the early phase of the second COVID-19 wave in Italy). The use of hydroxychloroquine, either as monotherapy or in combination with an antibiotic, entered in the top 10 therapies in the period between March 2020 and May 2020. The rank of NSAID therapy (as monotherapy or in combination with antibiotic) and that of vitamin D in monotherapy fluctuated by month.

## Discussion

This is the first study aiming to analyze the real practice drug use in COVID-19 patients in an outpatient setting taking into account EBM recommendations issued by a central competent authority. Although there is scarcity of data, most of these drug treatments were presumably prescribed assuming a positive impact on the COVID-19 illness. In this analysis, the use of drug therapy in the management of outpatient cases of COVID-19 was frequent, exceeding thirty percent of the cases in some months. This percentage is particularly high considering that most of the Italian COVID-19-positive patients in the study period were asymptomatic or with mild or moderate symptoms and did not require hospitalization (Epicentro- Istituto Superiore di Sanità, 2021). In this context, we were able to measure an important off-label use with unproven drug therapies.

In times of crisis such as the COVID-19 pandemic, it has been described that there is inclination of clinicians to prescribe unproven therapies, which may be potentially harmful, assuming they were beneficial for their patients (Payne et al., 2021). In a recent systematic review, Diaz-Arocutipa et al. (2021) showed a higher prevalence of QTc prolongation in COVID-19 patients treated with hydroxychloroquine/chloroquine alone or in combination with azithromycin in comparison to no treatment.

On the other hand, a significant reduction in general outpatient prescriptions issued during the COVID-19 period has been reported. In particular, an interrupted time series analysis on outpatient antibiotic prescriptions in Ontario, Canada, showed a 31.2% (95% CI, 27.0–35.1%) reduction in total antibiotic prescriptions in outpatient settings during the COVID-19 pandemic driven by less antibiotic prescription for respiratory indications and largely explained by decreased visits for respiratory infections (Kitano et al., 2021). Instead, some single-center retrospective chart reviews showed that during the first peak of COVID-19, in an area with high infection burden, there was an overall increase in antimicrobial prescription in outpatient primary care clinics (Douglas et al., 2021). According to these preliminary results focused on the outpatient COVID-19-positive population, we attempted to investigate drugs included in the official positive or negative recommendations issued by national regulatory authorities.

In the hindsight and according to our study, several phenomena can be identified. The percentage of COVID-19 outpatients on off-label drug therapies follows the pandemic wave trend. The most used outpatient therapy was based on antibiotic drugs, specifically azithromycin, despite a negative recommendation of the central competent authority. These data were also confirmed considering the combinations of corticosteroids and antithrombotics. On the other hand, the use of corticosteroids increased as reflection of several positive recommendations by the national regulatory agency.

The use of hydroxychloroquine in COVID-19 patients was limited to the early pandemic period where evidence on its potential benefit was controversial, while it disappeared after the publications of several negative recommendations. Antithrombotic therapy was widely used even if its prescription in the outpatient setting was recommended only in specific case. Another population-based study of outpatients with confirmed SARS-CoV-2 infection showed similar results to our analysis (Crisafulli et al., 2021). In particular, azithromycin and, to a slightly lower extent, glucocorticoids were widely used among patients testing positive for SARS-CoV-2, even if asymptomatic, in general practice, in contrast to the Italian Health Ministry recommendations.

Limitations of the study were that the analyzed pharmaceutical demand describes the National Health Service (NHS) burden in one single Italian region, which does not necessarily represent all the Italian outpatient COVID-19 drug treatments. Furthermore, out-of-pocket drug purchases might have an influence on the proportion of prescription of study’s drugs, in particular for NSAIDs and vitamin D. However, drug utilization studies like this one may help understand how the NHS was able to deal with the uncertainties regarding different repurposed drug therapies for COVID-19, even before the availability of evidence from randomized controlled trials. Finally, the indication for drug prescription is not known, so in some cases, the drug use could not be related to COVID-19 infection. To overcome this concern, we decided to consider only drug prescriptions in a window close to COVID-19 diagnosis taking into account both the possible delay in laboratory test result (3 days before diagnosis) and the possible disease progression (7 days after diagnosis). Moreover, for each exposure group, we replicated the analyses considering only incident users and results did not change (Supplementary Table S3).

Our study does not take into account how the availability of COVID-19 vaccines might have affected the results. However, only the frail population could be considered fully vaccinated during the study period, so we expected a slight reduction in the use of drug therapy in the management of COVID-19 outpatient cases.

Some authors presented the COVID-19 pandemic as a threat to traditional models of knowledge translation into clinical practice (Carley et al., 2020). Emergency forced the regulatory bodies to find new ways to promptly respond the uncertainty with experimental treatments based on spurious data. Off-label drug use without any control or appropriate protocol represents an avoidable health risk that needs to be handled. Furthermore, it is crucial to see how important is the role of the national competent authority in promoting and disseminating the correct and evidence-based information on the appropriate use of the drugs outside of the usual regulatory documents.

Further studies are needed to investigate factors that influenced EBM recommendations uptake in clinical practice, in order to make regulatory authority advices useful also in a contest of high uncertainty.

## Conclusion

In this study, we show a frequent use of drug therapy in the management of outpatient cases of COVID-19, mainly attributable to antibiotics use. Our research highlights the discrepancy between recommendations for care and clinical practice and the need for strategies to bridge gaps in evidence-informed decision-making.

## Data Availability

The data supporting the findings of this article are available at aggregated level from the authors upon reasonable request and with permission of Lazio region. Requests to access should be directed to corresponding author.
