# Supplementary material for: Drug Prescriptions in the Outpatient Management of COVID-19: Evidence-Based Recommendations Versus Real Practice
Source: Front Pharmacol. 2022 Mar 24;13:825479. doi: 10.3389/fphar.2022.825479 (PMC8988061; doi:10.3389/fphar.2022.825479)
Supplement: Supplementary file 1 [file DataSheet1.docx]

**Supplementary Material**

Table 1. Drug use prevalence and 95% CI among Covid-19 cases stratified by calendar month and drug class

|  | Mar 20 | Apr 20 | May 20 | Jun 20 | Jul 20 | Aug 20 | Sep 20 | Oct 20 | Nov 20 | Dec 20 | Jan 20 | Feb 21 | Mar 21 | Apr 21 | May 21 | Total |
| --- | --- | --- | --- | --- | --- | --- | --- | --- | --- | --- | --- | --- | --- | --- | --- | --- |
| N° Covid-19 cases | 2,842 | 2,472 | 642 | 307 | 417 | 2,880 | 5,079 | 38,402 | 73,298 | 42,347 | 38,416 | 27,450 | 49,217 | 34,311 | 13,624 | 331,704 |
| Antibiotics |  |  |  |  |  |  |  |  |  |  |  |  |  |  |  |  |
| *prevalence* | 14.7 | 9.2 | 8.6 | 5.5 | 7.9 | 8.8 | 12.1 | 18.7 | 17.8 | 22.4 | 24.5 | 30.1 | 31.3 | 26.4 | 21.3 | 23.0 |
| *IC-* | 13.4 | 8.0 | 6.4 | 3.0 | 5.3 | 7.8 | 11.2 | 18.3 | 17.5 | 22.0 | 24.1 | 29.6 | 30.9 | 25.9 | 20.6 | 22.9 |
| *IC+* | 16.0 | 10.3 | 10.7 | 8.1 | 10.5 | 9.9 | 13.0 | 19.1 | 18.1 | 22.8 | 24.9 | 30.7 | 31.7 | 26.8 | 22.0 | 23.2 |
| Antithrombotics |  |  |  |  |  |  |  |  |  |  |  |  |  |  |  |  |
| *prevalence* | 3.4 | 5.1 | 4.4 | 3.6 | 2.6 | 2.2 | 3.4 | 6.2 | 7.2 | 7.9 | 8.6 | 9.2 | 10.1 | 8.1 | 5.4 | 7.8 |
| *IC-* | 2.7 | 4.3 | 2.8 | 1.5 | 1.1 | 1.7 | 2.9 | 5.9 | 7.0 | 7.6 | 8.4 | 8.9 | 9.9 | 7.8 | 5.0 | 7.7 |
| *IC+* | 4.0 | 6.0 | 5.9 | 5.7 | 4.2 | 2.7 | 3.9 | 6.4 | 7.4 | 8.1 | 8.9 | 9.6 | 10.4 | 8.4 | 5.7 | 7.9 |
| Corticosteroids |  |  |  |  |  |  |  |  |  |  |  |  |  |  |  |  |
| *prevalence* | 2.3 | 1.9 | 2.5 | 2.0 | 2.2 | 1.7 | 3.8 | 10.1 | 11.7 | 15.1 | 16.9 | 21.3 | 20.9 | 16.0 | 12.1 | 14.8 |
| *IC-* | 1.7 | 1.4 | 1.3 | 0.4 | 0.8 | 1.3 | 3.3 | 9.8 | 11.5 | 14.8 | 16.6 | 20.8 | 20.5 | 15.6 | 11.6 | 14.7 |
| *IC+* | 2.8 | 2.4 | 3.7 | 3.5 | 3.6 | 2.2 | 4.3 | 10.4 | 11.9 | 15.4 | 17.3 | 21.8 | 21.3 | 16.4 | 12.7 | 14.9 |
| Vitamin D |  |  |  |  |  |  |  |  |  |  |  |  |  |  |  |  |
| *prevalence* | 0.6 | 0.9 | 0.9 | 0.3 | 0.2 | 0.2 | 0.7 | 0.7 | 0.9 | 0.9 | 1.1 | 1.2 | 1.3 | 1.2 | 1.0 | 1.0 |
| *IC-* | 0.3 | 0.5 | 0.2 | 0.0 | 0.0 | 0.0 | 0.5 | 0.7 | 0.8 | 0.8 | 1.0 | 1.1 | 1.2 | 1.1 | 0.9 | 1.0 |
| *IC+* | 0.8 | 1.3 | 1.7 | 1.0 | 0.7 | 0.4 | 1.0 | 0.8 | 0.9 | 1.0 | 1.2 | 1.3 | 1.4 | 1.3 | 1.2 | 1.0 |
| NSAIDs |  |  |  |  |  |  |  |  |  |  |  |  |  |  |  |  |
| *prevalence* | 0.8 | 1.0 | 0.9 | 0.3 | 1.7 | 0.7 | 1.0 | 1.0 | 1.1 | 1.8 | 2.4 | 2.7 | 3.4 | 4.4 | 4.2 | 2.2 |
| *IC-* | 0.5 | 0.6 | 0.2 | 0.0 | 0.4 | 0.4 | 0.7 | 0.9 | 1.0 | 1.7 | 2.2 | 2.5 | 3.2 | 4.2 | 3.9 | 2.2 |
| *IC+* | 1.1 | 1.4 | 1.7 | 1.0 | 2.9 | 1.0 | 1.3 | 1.1 | 1.1 | 1.9 | 2.5 | 2.9 | 3.6 | 4.7 | 4.6 | 2.3 |
| Hydroxychloroquine |  |  |  |  |  |  |  |  |  |  |  |  |  |  |  |  |
| *prevalence* | 2.2 | 1.7 | 2.0 | 0.0 | 0.2 | 0.3 | 0.2 | 0.2 | 0.1 | 0.1 | 0.1 | 0.1 | 0.1 | 0.1 | 0.1 | 0.2 |
| *IC-* | 1.6 | 1.2 | 0.9 | 0.0 | 0.0 | 0.1 | 0.1 | 0.1 | 0.1 | 0.1 | 0.1 | 0.1 | 0.1 | 0.1 | 0.0 | 0.1 |
| *IC+* | 2.7 | 2.2 | 3.1 | 0.0 | 0.7 | 0.6 | 0.3 | 0.2 | 0.1 | 0.1 | 0.2 | 0.2 | 0.2 | 0.1 | 0.1 | 0.2 |
| Oxygen |  |  |  |  |  |  |  |  |  |  |  |  |  |  |  |  |
| *prevalence* | 0.2 | 0.4 | 0.0 | 0.3 | 0.5 | 0.1 | 0.3 | 0.6 | 1.2 | 1.4 | 1.6 | 1.4 | 1.7 | 1.3 | 0.8 | 1.2 |
| *IC-* | 0.0 | 0.1 | 0.0 | 0.0 | 0.0 | 0.0 | 0.1 | 0.5 | 1.2 | 1.3 | 1.5 | 1.2 | 1.6 | 1.2 | 0.7 | 1.2 |
| *IC+* | 0.4 | 0.6 | 0.0 | 1.0 | 1.1 | 0.3 | 0.4 | 0.6 | 1.3 | 1.5 | 1.7 | 1.5 | 1.8 | 1.5 | 1.0 | 1.3 |

Table 2. The three most commonly used drugs in each category.

| Drug class | Drug | ATC | Covid-19 drug users in the study period | Percentage of users on drug category users |
| --- | --- | --- | --- | --- |
| Antibiotics | Azithromycin | J01FA10 | 52383 | 68.6% |
|  | Clarithromycin | J01FA09 | 9486 | 12.4% |
|  | Cefixime | J01DD08 | 5603 | 7.3% |
| Antithrombotics | Enoxaparin | B01AB05 | 20406 | 79.0% |
|  | [Acetylsalicylic acid](https://icdcode.info/atc-ddd/index-b01ac06.html) | B01AC06 | 3204 | 12.4% |
|  | Clopidogrel | B01AC04 | 702 | 2.7% |
| Corticosteroids | Prednisone | H02AB07 | 31332 | 63.9% |
|  | Betametasone | H02AB01 | 9507 | 19.4% |
|  | Dexametasone | H02AB02 | 5758 | 11.7% |
| NSAIDs | Ibruprofen | M01AE01 | 2681 | 36.0% |
|  | Nimesulide | M01AX17 | 1752 | 23.5% |
|  | Ketoprofen | M01AE03 | 1219 | 16.4% |
| Vitamin D | [Colecalciferol](https://www.whocc.no/atc_ddd_index/?code=A11CC05&showdescription=yes) | A11CC05 | 2571 | 76.9% |
|  | [Calcifediol](https://www.whocc.no/atc_ddd_index/?code=A11CC06&showdescription=yes) | A11CC06 | 346 | 10.4% |
|  | [Alfacalcidol](https://www.whocc.no/atc_ddd_index/?code=A11CC03&showdescription=yes) | A11CC03 | 231 | 6.9% |

Table 3. Drug use prevalence in new users* and 95% CI among Covid-19 cases stratified by calendar month and drug class

|  | Mar 20 | Apr 20 | May 20 | Jun 20 | Jul 20 | Aug 20 | Sep 20 | Oct 20 | Nov 20 | Dec 20 | Jan 20 | Feb 21 | Mar 21 | Apr 21 | May 21 | Total |
| --- | --- | --- | --- | --- | --- | --- | --- | --- | --- | --- | --- | --- | --- | --- | --- | --- |
| N° Covid-19 cases | 2,842 | 2,472 | 642 | 307 | 417 | 2,880 | 5,079 | 38,402 | 73,298 | 42,347 | 38,416 | 27,450 | 49,217 | 34,311 | 13,624 | 331,704 |
| Antibiotics |  |  |  |  |  |  |  |  |  |  |  |  |  |  |  |  |
| *prevalence* | 11.4 | 6.6 | 6.4 | 5.2 | 6.2 | 8.1 | 10.9 | 16.5 | 15.2 | 19.1 | 21.0 | 26.4 | 27.6 | 23.0 | 18.9 | 20.0 |
| *IC-* | 10.3 | 5.6 | 4.5 | 2.7 | 3.9 | 7.1 | 10.1 | 16.1 | 14.9 | 18.8 | 20.6 | 25.9 | 27.2 | 22.6 | 18.3 | 19.9 |
| *IC+* | 12.6 | 7.5 | 8.3 | 7.7 | 8.6 | 9.1 | 11.8 | 16.9 | 15.4 | 19.5 | 21.5 | 27.0 | 28.0 | 23.5 | 19.6 | 20.1 |
| Antithrombotics |  |  |  |  |  |  |  |  |  |  |  |  |  |  |  |  |
| *prevalence* | 0.9 | 2.6 | 2.5 | 1.6 | 1.4 | 1.5 | 2.2 | 4.4 | 4.7 | 5.0 | 5.5 | 6.5 | 7.7 | 5.9 | 3.8 | 5.3 |
| *IC-* | 0.5 | 2.0 | 1.3 | 0.2 | 0.3 | 1.1 | 1.8 | 4.2 | 4.5 | 4.8 | 5.3 | 6.2 | 7.4 | 5.6 | 3.5 | 5.3 |
| *IC+* | 1.2 | 3.3 | 3.7 | 3.0 | 2.6 | 2.0 | 2.7 | 4.6 | 4.8 | 5.2 | 5.7 | 6.8 | 7.9 | 6.1 | 4.1 | 5.4 |
| Corticosteroids |  |  |  |  |  |  |  |  |  |  |  |  |  |  |  |  |
| *prevalence* | 1.9 | 1.4 | 2.3 | 1.6 | 2.2 | 1.6 | 3.6 | 9.6 | 10.9 | 14.2 | 15.9 | 20.4 | 19.9 | 15.1 | 11.5 | 14.0 |
| *IC-* | 1.4 | 1.0 | 1.2 | 0.2 | 0.8 | 1.2 | 3.1 | 9.3 | 10.7 | 13.8 | 15.5 | 19.9 | 19.6 | 14.7 | 11.0 | 13.8 |
| *IC+* | 2.4 | 1.9 | 3.5 | 3.0 | 3.6 | 2.1 | 4.1 | 9.9 | 11.2 | 14.5 | 16.3 | 20.9 | 20.3 | 15.5 | 12.0 | 14.1 |
| Vitamin D |  |  |  |  |  |  |  |  |  |  |  |  |  |  |  |  |
| *prevalence* | 0.2 | 0.5 | 0.6 | 0.3 | 0.0 | 0.1 | 0.4 | 0.5 | 0.5 | 0.5 | 0.7 | 0.8 | 0.9 | 0.8 | 0.7 | 0.6 |
| *IC-* | 0.0 | 0.2 | 0.0 | -0.3 | 0.0 | 0.0 | 0.3 | 0.4 | 0.5 | 0.4 | 0.6 | 0.7 | 0.8 | 0.7 | 0.6 | 0.6 |
| *IC+* | 0.4 | 0.8 | 1.2 | 1.0 | 0.0 | 0.3 | 0.6 | 0.6 | 0.6 | 0.6 | 0.8 | 0.9 | 1.0 | 0.9 | 0.9 | 0.7 |
| NSAIDs |  |  |  |  |  |  |  |  |  |  |  |  |  |  |  |  |
| *prevalence* | 0.6 | 0.8 | 0.8 | 0.0 | 1.2 | 0.4 | 0.7 | 0.7 | 0.8 | 1.5 | 1.9 | 2.3 | 2.8 | 3.8 | 3.5 | 1.8 |
| *IC-* | 0.3 | 0.4 | 0.1 | 0.0 | 0.2 | 0.2 | 0.5 | 0.7 | 0.7 | 1.3 | 1.8 | 2.1 | 2.7 | 3.6 | 3.2 | 1.8 |
| *IC+* | 0.8 | 1.1 | 1.5 | 0.0 | 2.2 | 0.7 | 1.0 | 0.8 | 0.8 | 1.6 | 2.0 | 2.4 | 3.0 | 4.0 | 3.8 | 1.9 |
| Hydroxychloroquine |  |  |  |  |  |  |  |  |  |  |  |  |  |  |  |  |
| *prevalence* | 2.1 | 1.6 | 2.0 | 0.0 | 0.2 | 0.3 | 0.2 | 0.1 | 0.1 | 0.1 | 0.1 | 0.1 | 0.1 | 0.1 | 0.0 | 0.1 |
| *IC-* | 1.6 | 1.1 | 0.9 | 0.0 | 0.0 | 0.1 | 0.1 | 0.1 | 0.1 | 0.0 | 0.1 | 0.1 | 0.1 | 0.1 | 0.0 | 0.1 |
| *IC+* | 2.6 | 2.1 | 3.1 | 0.0 | 0.7 | 0.6 | 0.3 | 0.2 | 0.1 | 0.1 | 0.1 | 0.1 | 0.2 | 0.1 | 0.1 | 0.1 |
| Oxygen |  |  |  |  |  |  |  |  |  |  |  |  |  |  |  |  |
| *prevalence* | 0.1 | 0.2 | 0.0 | 0.0 | 0.0 | 0.1 | 0.2 | 0.5 | 1.1 | 1.2 | 1.5 | 1.3 | 1.6 | 1.3 | 0.8 | 1.1 |
| *IC-* | 0.0 | 0.0 | 0.0 | 0.0 | 0.0 | 0.0 | 0.0 | 0.4 | 1.0 | 1.1 | 1.4 | 1.2 | 1.5 | 1.2 | 0.6 | 1.1 |
| *IC+* | 0.2 | 0.4 | 0.0 | 0.0 | 0.0 | 0.2 | 0.3 | 0.5 | 1.2 | 1.3 | 1.6 | 1.4 | 1.8 | 1.4 | 0.9 | 1.2 |

New users were defined excluding user of drug class with a previous use from 15 to the 90 days before Covid-19 diagnosis

STROBE Statement—checklist of items that should be included in reports of observational studies

|  | Item No. | Recommendation | Page  No. |  |
| --- | --- | --- | --- | --- |
| **Title and abstract** | 1 | (*a*) Indicate the study’s design with a commonly used term in the title or the abstract | 1 |  |
|  |  | (*b*) Provide in the abstract an informative and balanced summary of what was done and what was found | 2 |  |
| Introduction | | | |  |
| Background/rationale | 2 | Explain the scientific background and rationale for the investigation being reported | 3 |  |
| Objectives | 3 | State specific objectives, including any prespecified hypotheses | 3 |  |
| Methods | | | |  |
| Study design | 4 | Present key elements of study design early in the paper | 4 |  |
| Setting | 5 | Describe the setting, locations, and relevant dates, including periods of recruitment, exposure, follow-up, and data collection | 4 |  |
| Participants | 6 | (*a*) Give the eligibility criteria, and the sources and methods of selection of participants | 4 |  |
|  |  |  |  |  |
| Variables | 7 | Clearly define all outcomes, exposures, predictors, potential confounders, and effect modifiers. Give diagnostic criteria, if applicable | 4 |  |
| Data sources/ measurement | 8* | For each variable of interest, give sources of data and details of methods of assessment (measurement). Describe comparability of assessment methods if there is more than one group | 4 |  |
| Bias | 9 | Describe any efforts to address potential sources of bias | 6/7 |  |
| Study size | 10 | Explain how the study size was arrived at | 5 |  |

| Quantitative variables | | 11 | | Explain how quantitative variables were handled in the analyses. If applicable, describe which groupings were chosen and why | 5 | |  |
| --- | --- | --- | --- | --- | --- | --- | --- |
| Statistical methods | | 12 | | (*a*) Describe all statistical methods, including those used to control for confounding | 4 | |  |
|  |  |  |  | (*b*) Describe any methods used to examine subgroups and interactions | 4 | |  |
|  |  |  |  | (*c*) Explain how missing data were addressed |  | |  |
|  |  |  |  | (*d*) If applicable, describe analytical methods taking account of sampling strategy | n.a. | |  |
|  |  |  |  | (*e*) Describe any sensitivity analyses | 7 | |  |
| Results | | | | | | | |
| Participants | | 13* | | (a) Report numbers of individuals at each stage of study—eg numbers potentially eligible, examined for eligibility, confirmed eligible, included in the study, completing follow-up, and analysed | 5 | |  |
|  |  |  |  | (b) Give reasons for non-participation at each stage | 5 | |  |
|  |  |  |  | (c) Consider use of a flow diagram |  | |  |
| Descriptive data | | 14* | | (a) Give characteristics of study participants (eg demographic, clinical, social) and information on exposures and potential confounders | 5 | |  |
|  |  |  |  | (b) Indicate number of participants with missing data for each variable of interest | 5 | |  |
|  |  |  |  | (c) *Cohort study*—Summarise follow-up time (eg, average and total amount) | n.a. | |  |
| Outcome data | | 15* | | Report numbers of outcome events or summary measures | n.a. | |  |
| Main results | | 16 | | (*a*) Give unadjusted estimates and, if applicable, confounder-adjusted estimates and their precision (eg, 95% confidence interval). Make clear which confounders were adjusted for and why they were included | n.a. | |  |
|  |  |  |  | (*b*) Report category boundaries when continuous variables were categorized | n.a. | |  |
|  |  |  |  | (*c*) If relevant, consider translating estimates of relative risk into absolute risk for a meaningful time period | n.a. | |  |
| Other analyses | 17 | | Report other analyses done—eg analyses of subgroups and interactions, and sensitivity analyses | | 7 |  | |
| Discussion | | | | | | | |
| Key results | 18 | | Summarise key results with reference to study objectives | | 7 |  | |
| Limitations | 19 | | Discuss limitations of the study, taking into account sources of potential bias or imprecision. Discuss both direction and magnitude of any potential bias | | 7 |  | |
| Interpretation | 20 | | Give a cautious overall interpretation of results considering objectives, limitations, multiplicity of analyses, results from similar studies, and other relevant evidence | | 7/8 |  | |
| Generalisability | 21 | | Discuss the generalisability (external validity) of the study results | | 7 |  | |
| Other information | | |  | | | | |
| Funding | 22 | | Give the source of funding and the role of the funders for the present study and, if applicable, for the original study on which the present article is based | | 9 |  | |
